# The impact of green finance and local regulations on industrial green innovation efficiency in China

**DOI:** 10.1007/s11356-023-31314-w

**Published:** 2023-12-05

**Authors:** YiHan Xia, LuYi Luo, KaiWen Ji, Chao Huang, Fei Wan, ZhiGang Wang

**Affiliations:** 1 Management Science and Engineering Research Center, Jiangxi Normal University, Nanchang, 330022 Jiangxi China; 2 The School of Economics, Jiangxi University of Finance and Economics, Nanchang, 330013 Jiangxi China; 3 School of Political Science and Law, Jiangxi Normal University, Nanchang, 330022 Jiangxi China; 4 China Galaxy Securities Co., Ltd., Shanghai Branch, Shanghai, 200129 China; 5 Jiangxi Jiangtou Capital Holding Co., Ltd., Nanchang, 330000 Jiangxi China

**Keywords:** Green finance, Green technology innovation, Environmental regulation, Moderation effect

## Abstract

When the incentive mechanism of green finance fails to fully promote green technology innovation in industrial enterprises, local government environmental regulations become an important tool in correcting this market failure. However, due to the “follow the cost” hypothesis, the moderating effect of the local government environmental regulation is heterogeneous. In order to explore the impact mechanism of green finance development on the efficiency of green technology innovation in industrial enterprises, spatial effects as well as the heterogeneous moderating effect of local government environmental regulation, this paper systematically evaluates the development level of green finance in 30 provinces in China from 2009 to 2019. It estimates the efficiency of green technology innovation in industrial enterprises using the super-efficiency SBM model, and empirically analyzes the impact mechanism and moderating effect using the spatial Durbin model. The results show that: (1) green finance not only positively impacts the efficiency improvement of green technology innovation in industrial enterprises but also has significant spatial spillover effects; (2) local government environmental regulation has a nonlinear “inverted U-shaped” moderating effect between the green finance development and the efficiency of green technology innovation in industrial enterprises. Based on the research conclusions, this paper proposes policy recommendations from the perspectives of deepening the regional connectivity of green finance and promoting joint regulation by local governments.

## Introduction

The Chinese government recognizes that promoting green and low-carbon economic and social development is essential to achieving high-quality development. Compared with developed countries that have already achieved industrialization, the proportion of traditional high-energy-consuming industries in China’s industry is still relatively high. Industrial enterprises have not completely overcome the development model dilemma of “high input, high consumption, and high emissions,” as the fundamental reason lies in the uneven development of regional innovation capabilities across the country (Wei et al. 2020). According to the China’s Second National Pollutant Source Census Report, industrial pollution sources account for almost 70% of all pollution sources in China. Therefore, it is necessary to further promote the green and high-quality development of industry. In particular, the proposal of the “peak carbon dioxide emissions” and “carbon neutrality” goals indicates that the trend toward clean and low-carbon technology innovation for traditional industries is imminent. The comprehensive low-carbon transformation of industrial enterprises necessitates significant green investment and financing. Green finance can fundamentally solve the funding gap and financing bottleneck brought about by innovation, and provide guidance and assurance for green technology innovation. The “14th Five-Year Plan for Industrial Green Development” by the Ministry of Industry and Information Technology clearly defines the main goals of industrial green and low-carbon development during the 14th Five-Year Plan period and emphasizes the importance of green finance in supporting industrial green development. Therefore, exploring the impact of green finance on the efficiency of industrial green technology innovation has become crucial in China’s promotion of the “peak carbon dioxide emissions” and “carbon neutrality” goals in the industrial sector.

Internationally, green finance refers to the integration of environmental protection and economic benefits by financial institutions and the prioritization of investment in various environmental protection activities to promote the transformation of the green economy (Zheng et al. 2021). According to the European Commission’s definition of green finance, it involves investment decisions that incorporate environmental, social, and governance principles to enhance the economic, social, and environmental performance of the monetary system.^1^ In practice, green finance is mainly divided into two categories in various countries: the first is voluntarily organized by financial institutions under the guidance of the Equator Principles and involves cautious investigation of the environmental and social issues of large-scale financing projects to promote positive environmental protection impacts (Macve and Chen 2010); the second is jointly participated in by central banks and governments to incorporate climate change-related risks into the operation of the financial system, forming the Network of Central Banks and Supervisors for Greening the Financial System (NGFS) (Campiglio et al. 2018). However, according to Dikau and Volz (2021) survey of 135 central banks worldwide, only 12% of central banks in the sample they surveyed included sustainable development as a development goal, and due to conflicts between government policy goals and central bank macroeconomic regulation goals, the participation of central banks in green finance under the premise of maintaining independence from the government may result in policy distortion effects, and some people in some countries have questioned and expressed hidden concerns about the involvement of central banks in environmental regulation. Some studies have shown that although the independence of some foreign central banks leads to a series of risks associated with intervention in environmental policy, mitigating climate change is also one of the responsible goals of central banks (Campiglio et al. 2018). In China, as the central bank is one of the departments under the State Council and is under the jurisdiction of the central government, there is no conflict between policy goals and central bank macroeconomic regulation goals in the implementation of green finance policies, giving China institutional and regulatory advantages. At the same time, compared to the voluntary constraints of the industry under the Equator Principles, China’s green finance development has policy enforceability and exploitativeness. As the first country in the world to formulate a top-level design for green finance, China has organically combined the government’s environmental regulatory functions with the market’s internal driving force, initially established a green finance standard system, and the pilot experience of green finance reform and innovation zones is being promoted nationwide. China’s green finance mainly includes green credit, green bonds, green funds, green insurance, carbon finance, and other green investment and financing services that support environmental benefit improvement, promoting the coordinated development of green finance at different levels in recent years. At the same time, supporting local government environmental regulations can internalize external environmental costs to financial institutions and industrial enterprises, serving as a “bridge” for the implementation of green finance policies and the green transformation strategy of industrial enterprises, and to some extent, opening up the channel between green finance and industrial enterprises. However, local governments may weaken the constraint mechanism on non-green industrial enterprises due to economic performance considerations. They which could undermine the incentive path for green technology innovation in industrial enterprises promoted by the development of green finance (Zhang et al. 2019). How to coordinate the development of green finance, local government environmental regulations, and industrial enterprise green technology innovation, promote the efficiency improvement of industrial enterprise green technology innovation, and achieve coordinated regional innovation capability development has become a pressing issue that needs to be addressed in the current push for the comprehensive green transformation of the economy and society.

Current research mainly focuses on the incentive effects of certain green finance services. For example, Lin et al. (2018) used environmental protection companies as an example and discovered that direct financing represented by green securities is more effective for corporate development than indirect financing represented by green loans. Flammer (2021) found that the issuance of green bonds, based on global data, can promote corporate green innovation. Wang Xin and Wang Ying (2021) found that domestic green loan policies can promote green innovation in industries with green loan restrictions by reducing agency costs and improving investment efficiency, while Niu et al. (2020) found from the perspective of financing costs that green loan policies to some extent solve the problem of financing difficulties for green projects. Based on the above research, it is easy to find that various types of green finance services can have a positively impact on corporate green innovation. As a policy-driven financial service, green finance not only needs bidirectional regulation by local government environmental regulations, but also needs to resolve the contradiction between profitability and public welfare in the implementation of green finance, and green finance products also need to innovate from both the supply and demand sides and build a financial service system that supports green technology innovation (Ma et al. 2020). Nevertheless, the green finance system in China still needs to be improved. Most of the green investments in the existing capital market still cannot achieve the “dual objectives” of financial and environmental performance coordination (Wei and Shu 2021), and the incentive effect of the green finance system cannot be directly reflected for now. For example, research conducted by Yin et al. (2021) found a “U”-shaped relationship between China’s green finance and green total factor productivity. From the above literature, most studies have examined the incentive effects of a particular financial service on corporate innovation, and there are concerns regarding the incentive ability of China’s green finance development system in the context of the actual situation of green finance development. However, there exists little existing research that directly explores the impact mechanism of the overall development level of green finance on industrial enterprise green technology innovation, particularly ignoring the regulatory mechanism of the impact of green finance under local government environmental regulations. Therefore, this article mainly considers the following questions: What is the impact mechanism of green finance development on industrial enterprise green technology innovation? Is there a spatial spillover effect at the national level? Can the regulatory role of local government environmental regulations deepen the impact of green finance development on industrial enterprise green technology innovation? Does the regulatory role of local government environmental regulations show heterogeneity?

The main contributions of this paper are as follows: (1) while existing research mostly explores the relationship between a specific green financial service and social green innovation, this paper goes further to investigate the impact of the overall development level of green finance on the efficiency of green technology innovation in industrial enterprises in China (You & Ouyang 2020), taking into account the significant spatial effects of green technology innovation and it explores whether there is a direct spatial effect and spatial spillover effect of the development level of green finance on technological innovation in industrial enterprises. (2) Green finance, as policy-oriented finance implemented from top to bottom by the central government, has not been fully considered for the regulatory effect of local government environmental regulations in most existing studies. Therefore, this paper selects local government environmental regulations as a moderating variable to explore the transmission mechanism of green finance development to green technology innovation in industrial enterprises under different intensities of environmental regulation.

## Theoretical analysis and research hypotheses

### Green finance and the green technology innovation of industrial enterprises

The fundamental driving force for the green transformation of industrial enterprises lies in green technology innovation (Yang et al. 2020). However, due to the dual externalities of technology and environment that green technology innovation possesses (Rennings 2000), i.e., green technology cannot be exclusively enjoyed by innovative enterprises and provide positive externalities for other polluting enterprises while the negative externalities of the environment make industrial enterprises more inclined to adopt backward non-green technologies under weak regulation. Based on innovation diffusion theory, green finance can expedite the diffusion of green technologies by lowering the cost of capital for enterprises (D’Orazio & Valente 2019). Lower financing costs make it more attractive for firms to adopt and implement green innovations, resulting in quicker technology diffusion and wider adoption within the industry. Meanwhile, resource dependency theory posits that an efficient green finance system provides capital support to enterprises, empowering them to engage more effectively in environmental innovation. Green finance, as a critical resource, enables businesses to access green financing through various means such as green bonds or loans. This facilitates investment in research and development, acquisition of green technologies, and the establishment of sustainable practices, thereby enhancing their competitive advantage in the green sector (Najaf & Najaf 2021). The implementation of green finance in China itself is based on serving the development of green industries and the transformation of traditional industries to green ones, resolving investment and financing constraints for industrial enterprises’ green technology innovation by regulating funds and promoting credit restructuring and green investment guidelines. From the perspective of the regulatory role of green finance, it provides an important external source of financing for green technology innovation for industrial enterprises while effectively resolving enterprise maturity mismatch risks and other liquidity risks. Additionally, it encourages backward capacity industries to innovate green technologies by influencing their financing costs and providing endogenous motivation for green technology transformation (Wang et al. 2021). From the perspective of the policy guidance role of green finance, it guides enterprise technology innovation direction, eliminates financing sources for backward capacity (Xie and Zhang 2021), and promotes efficient resource flow into green projects, playing a guiding role in the green transformation of industrial enterprises. From the perspective of the consulting role of green finance, by participating in the formulation of regional green transformation development plans and the implementation of financing schemes, it provides corresponding financial settlement services and project traction for the green technology innovation of industrial enterprises, improving the efficiency of green technology innovation and green fund utilization (Cheng et al. 2020). Based on this, the hypothesis is proposed:

- Hypotheses 1: The development of green finance has a positive impact on the green technology innovation of industrial enterprises.

The green technology innovation of industrial enterprises has a significant positive spatial agglomeration effect (You & Ouyang 2020). When industrial enterprises invest in green technologies, they often develop expertise and knowledge that can spill over to other firms and regions. This can foster a spatial diffusion of green innovation and accelerate the adoption of green technologies across a broader geographical area. Green finance can incentivize industrial enterprises to cluster together in areas where green technology innovation is encouraged and supported (Li and Gan 2021). By concentrating green innovation activities, these enterprises can share knowledge, resources, and specialized labor, leading to more efficient innovation processes and a higher likelihood of success in developing and adopting green technologies. Green finance plays a crucial role in facilitating the flow of green resources across regions through its fund regulation function, and it also promotes the transformation of industrial enterprises toward green technology in local and surrounding areas through its policy guidance function. Green finance can be instrumental in creating positive spatial spillovers, where the benefits of green technology innovation extend beyond the immediate enterprise and region. At the same time, as the implementation of green finance in China is relatively new, and green finance pilot projects are still being orderly rolled out in different regions, the social network space supported by green finance is not yet fully established (Dong & Nian 2020). Therefore, the implementation of green finance policies has significant regional differences. In the context of heterogeneous green innovation demands and the implementation of green finance policies in different regions, the impact of green finance development on the green technology innovation of industrial enterprises will also exhibit significant regional characteristics. Based on this, the following hypothesis is proposed:

- Hypotheses 2: The development of green finance has spatial effects on the green technology innovation of industrial enterprises.

### Green finance, environmental regulation, and green technology innovation of industrial enterprises

Due to the problem of adverse selection and moral hazard in the green finance market, the incentive mechanism of green finance cannot be fully utilized (Wang 2021). Environmental regulation is an important means to correct the failure of the green finance market (Li 2017), playing a normative and guiding role in the fund regulation process of green finance (Sun & Lu 2021). The implementation effect of green finance policies is also affected by the differences in local government environmental regulation (Jin & Mengqi 2011). Therefore, local government regulatory departments need to proactively regulate the business implementation and control capabilities of green finance entities and strengthen the constraint mechanism of green finance. In China, local governments have issued corresponding incentive and regulatory policies to improve the green finance system, such as setting up special funds for green finance and strengthening the comprehensive evaluation of local green finance development, which have played a positive guiding role in promoting green finance development. At the same time, the implementation of green finance policies will restrict “two high” (i.e., high-polluting and high-energy-consuming) enterprises from obtaining green investment. The weak environmental regulation based on policy incentives to a certain extent provides policy support for restricted enterprises (Zhang et al. 2017), thereby overcoming the green finance constraints of corresponding industrial enterprises and positively regulating the promoting effect of green finance on the green technology innovation of industrial enterprises.

The success of regional green technology innovation relies on the intensive utilization of input factors, and can form a positive feedback spatial agglomeration effect through the optimization of policy environment and demand-induced effects (Ji et al. 2020). According to Porter’s hypothesis, in a dynamic competitive environment, government environmental regulations can reduce production costs by promoting firms’ advantage productivity, regulating the endogenous driving forces of industrial firms’ green technology innovation, and forcing industrial firms to innovate in green technologies (Porter & Linde 1995), resulting in innovation compensation effects. However, classical economic theory suggests that formal environmental regulations can lead to an increase in production costs and a decrease in profits simultaneously, and severe environmental regulations can have a crowding-out effect on green technology innovation by industrial firms (Yu et al. 2020; Kang & Ru 2020). In view of the heterogeneity of local government environmental regulations, scholars have found different degrees of non-linear relationship in the role of environmental regulations from the perspectives of industrial sector green innovation efficiency (Xu & Li 2018), green technology progress (Zhang et al. 2021; Dong & Wang 2019), green competitiveness (Du et al. 2019), etc. Therefore, the following hypothesis is proposed:

- Hypotheses 3: The influence of green finance on the green technology innovation of industrial enterprises is limited by the non-linear regulatory role of local government environmental regulations.

## Research design

### Index system selection and measurement

#### Green finance development level

Combining with the status of green finance development in China and based on existing research, we constructed a provincial-level green finance development index system, which includes five primary indicators and eight secondary indicators, as shown in Table 1. However, the statistical scope of existing green credit balance in China is limited to the banking sector, and only a few provinces have published their green credit balance with different statistical methods. Therefore, based on the calculation methods proposed by Li et al. (2019) and Zhang and Dou (2020), we estimated the green credit balance of each bank in each province according to the proportion of its branch network scale to the national level, and then summed them up as the annual green credit balance of each province. The interest expenses of high-energy-consumption industries were calculated for the chemical raw material and chemical product manufacturing industry, the ferrous metal smelting and rolling processing industry, the non-ferrous metal smelting and rolling processing industry, the non-metallic mineral product industry, the petroleum processing, coking and nuclear fuel processing industry, and the production and supply of electricity and heat. We selected industrial enterprises that applied environmental protection and energy conservation technologies according to the “Guidance Catalogue for Green Industry” published by the National Development and Reform Commission. As there is a lack of statistics on environmental liability insurance, and agriculture is greatly influenced by natural environmental factors, we used the development of agricultural insurance as a proxy variable for green insurance, referring to the research of Fang and Lin (2019) and Yin et al. (2021).

**Table 1:** The evaluation index system of green finance development level

Primary indicators	Secondary indicators	Definition of the indicators	References	Indicator direction
Green loans	Green loans input	Green loan balance/gross loan portfolio	Li et al. (2019); Zhang & Dou (2020)	Positive
	Interest expenses of high-energy-consuming industries as a percentage of their industrial interest expenses	Interest expenses of high-energy-consuming industries/industrial interest expenses	Fang & Lin (2019); Liu & Wen (2019)	Negative
Green securities	Market value of green stocks as a percentage of total A-share market capitalization	Market value of environmental protection enterprises/total market value of A shares	Fang & Lin (2019); Han (2020)	Positive
	Level of green bond issuance	Regional green bond issuance amount/total national green bond issuance amount	Gao & Li (2021)	Positive
Green insurance	Agricultural insurance scale ratio	Agricultural insurance expenditure/agricultural insurance income	Fang & Lin (2019); Yin et al. (2021)	Positive
	Agricultural insurance claims payout ratio	Agricultural insurance expenditure/total insurance expenditure		Positive
Green investments	Pollution control investment ratio	Pollution control investment/GDP	Li et al. (2019)	Positive
Carbon finance	Carbon emission intensity	Carbon emissions/loan balance	Zhang & Zhang (2015); Yin et al. (2021)	Negative

This article combines subjective and objective weighting methods to determine the level of green finance development in each province from 2009 to 2019. The subjective weight adopts the expert rating method, which is a common practice in domestic literature, and mainly uses the rating coefficients in Li Xiaoxi’s “China Green Finance Report” (Li et al. 2014), with appropriate adjustments. The objective weight adopts the entropy method. Finally, the minimum information entropy principle is used to comprehensively evaluate the subjective and objective weights and reduce deviations. The weights calculated by the entropy method and expert rating method are denoted as and, ${w}_{1j} \mathrm{and }{w}_{2j}$ respectively, with specific formulas as follows:

1 $${w}_{j}=\frac{{\left({w}_{1j}\times {w}_{2j}\right)}^\frac{1}{2}}{{\sum }_{1}^{n}{\left({w}_{1j}\times {w}_{2j}\right)}^\frac{1}{2}}$$

The comprehensive weights of the evaluation index system for green finance development are shown in Table 2. Based on the comprehensive weights, the level of green finance development (GF) in each province is finally calculated:

**Table 2:** Weighting coefficients of the evaluation index system for green finance development

Primary indicators	Objective weight	Subjective weight	Comprehensive weight	Indicator level	Objective weight	Subjective weight	Comprehensive weight
Green loans	0.38	0.50	0.46	Level of green credit investment	0.21	0.25	0.24
				Proportion of interest payments by high energy-consuming industries	0.17	0.25	0.22
Green securities	0.32	0.3	0.31	Proportion of green stock market capitalization	0.26	0.15	0.21
				Level of green bond issuance	0.06	0.15	0.10
Green insurance	0.11	0.1	0.10	Ratio of agricultural insurance scale to agricultural insurance payout	0.09	0.05	0.07
				Agricultural insurance claim payout ratio	0.02	0.05	0.03
Green investments	0.16	0.05	0.10	Ratio of pollution control investment to GDP	0.16	0.05	0.10
Carbon finance	0.02	0.05	0.04	Carbon emissions intensity	0.02	0.05	0.04

2 $${GF}_{ij}=\sum\nolimits_{j=1}^nw_jx_{ij}^{'}$$

#### Efficiency of green technology innovation in industrial enterprises

This article presents a comprehensive measurement the efficiency of green technology innovation in industrial enterprises from three levels: scientific and technological input, scientific and technological output, and unexpected output, as shown in Table 3. Among them, due to the lack of updated data on industrial wastewater discharge in the “China Environmental Statistics Yearbook” after 2015, so the chemical oxygen demand (COD) in industrial wastewater is used as a proxy variable for water pollution; the amount of industrial solid waste emissions is obtained by subtracting the comprehensive utilization of industrial solid waste from the amount of industrial solid waste generated.

**Table 3:** Assessment index system for green technology innovation efficiency of industrial enterprises

Measurement category	Indicator name	Indicator definition
Input variables	Research and development personnel input	Full-time equivalent of R&D personnel in industrial enterprises above designated size
	Research and development capital input	Accumulated internal R&D expenditure of industrial enterprises above designated size
Expected output variables	New product output	Sales revenue of new products
	Invention patent output	Number of invention patent applications
Unexpected output variables	Atmospheric pollution	Industrial sulfur dioxide emissions
	Water pollution	Chemical oxygen demand (COD) emissions in industrial wastewater
	Solid waste pollution	Industrial solid waste discharge

Firstly, the non-radial non-angular SBM model that considers non-expected output is used to evaluate the green technological innovation efficiency of industrial enterprises under variable scale returns in each province from 2009 to 2019. The evaluation results reveal that Anhui, Guangdong, Hainan, Shanghai, Tianjin, Zhejiang, and other provinces have achieved efficient status in terms of efficiency. To further differentiate effective units and accurately grasp the differences in regional industrial green technological innovation efficiency among provinces in China, the super-efficiency SBM model is introduced (Tone 2001; Tone & Tsutsui 2010) based on the original model to further differentiate effective units and calculate the green technological innovation efficiency (GTIE) of industrial enterprises. The specific formula is as follows:

3 $$\begin{array}{c}\mathrm{min}\theta =\frac{1+\frac{1}{m}\sum_{i=1}^{m}{s}_{i}^{-}/{x}_{ik}}{1-\frac{1}{s}\sum_{r=1}^{s}{s}_{r}^{+}/{y}_{rk}}\\ s.t.\sum_{j=1,j\ne k}{x}_{ij}{\lambda }_{j}-{s}_{i}^{-}\le {x}_{ik}\left(i=\mathrm{1,2},\dots ,m\right)\\ \sum_{j=1,j\ne k}{y}_{rj}{\lambda }_{j}+{s}_{r}^{+}\ge {y}_{rk}\left(r=\mathrm{1,2},\dots ,s\right)\\ {\lambda }_{j}\ge 0,j=\mathrm{1,2},\dots ,n\left(j\ne k\right), {s}_{i}^{-}\ge 0, {s}_{r}^{+}\ge 0\end{array}$$

#### Local government environmental regulation

The commonly used method to measure government environmental regulation is by calculating the investment made in industrial pollution control in relation to the total industrial output value. However, in addition to examining the government’s investment in pollution control, local government environmental regulation also needs to be tailored to the local industrial structure. Therefore, this paper refers to the method proposed by Song et al. (2019) to construct the evaluation index system for local government environmental regulation. The construction of this index is divided into the following steps:

Firstly, the proportion of pollution control investment in industrial output value in each province is calculated as follows:

4 $${{\mathrm{FERI}}_{it}}^{*}=\frac{{E}_{it}}{{Y}_{it}}$$

${{\mathrm{FERI}}_{it}}^{*}$ represents the ratio of pollution control investment to industrial output value in province *i* and year *t*, ${E}_{it}$ represents the amount of industrial pollution control investment completed in province *i* and year *t*, and ${Y}_{it}$ represents the total industrial output value in province *i* and year* t*.

Secondly, we calculate the industrial structure ratio of each province each year. The industrial structure varies among provinces, and the environmental regulation intensity of local governments will be overestimated in provinces with heavy polluting industries, while it may be underestimated in provinces with clean and environmentally friendly industries. The industrial structure ratio is defined as:

5 $${\mathrm{FERI}}_{it}=\frac{{Y}_{it}}{{\mathrm{GDP}}_{it}}$$

${\mathrm{FERI}}_{it}$ represents the industrial structure of province $i$ in year *t*, and ${\mathrm{GDP}}_{it}$ represents the local production value of province $i$ in year *t*.

Finally, we use the industrial structure to adjust the intensity of local government environmental regulation, and the local government environmental regulation (ER) is defined as:

6 $${\mathrm{ER}}_{it}=\frac{{{\mathrm{FERI}}_{it}}^{*}}{{\mathrm{FERI}}_{it}}\times 100$$

The larger the ${\mathrm{ER}}_{it}$ value, the greater the strength of local government’s environmental regulation; conversely, a weaker value indicates weaker regulation.

#### Control variables

Green technology innovation in industrial enterprises is influenced by various factors, including but not limited to green finance. The degree of openness, government investment in science and technology, and foreign direct investment are also significant factors. Referring to relevant research, this article selects the following control variables: level of openness (OPEN), represented by the total import and export volume/regional GDP; research and development investment (TS), represented by government investment in science and technology/local fiscal expenditure; level of foreign direct investment (FDI), represented by foreign direct investment/regional GDP.

#### Data source

The data for measuring the level of green finance development indicators were collected from sources such as the *CSMAR database*, *China Industrial Statistics Yearbook*, *Wind database*, *China Environmental Statistics Yearbook*, *China Insurance Yearbook*, and statistical yearbooks of various provinces over the years. The data for measuring industrial enterprise green technology innovation efficiency were obtained from sources such as the *China Science and Technology Statistics Yearbook*, *China Energy Statistics Yearbook*, and *China Environmental Statistics Yearbook*. The data for measuring local government environmental regulation were collected from sources such as *China Industrial Statistics Yearbook*, *China Environmental Statistics Yearbook*, and statistical yearbooks of various provinces over the years. The control variable data were obtained from sources such as the *China Science and Technology Statistics Yearbook*, statistical yearbooks of various provinces over the years, and statistical bulletins. Due to severe data missingness in Tibet, this paper excluded Tibet and used 30 provinces as research objects. In addition, given the high degree of deviation in data caused by the occurrence of the COVID-19 pandemic from 2020 to 2022, this paper excluded the data from these 3 years and used the data from 2009 to 2019 as the sample.

#### Descriptive statistics

Table 4 offers the descriptive statistics of the above variables. The result shows that average the efficiency of green technology innovation in industrial enterprises (GTIE) is 0.617, indicating most efficiency of green technology innovation of industrial enterprises in most Chinese provinces does not reach the efficiency effective. The average level of local government environmental regulation (ER) is 0.377, with a moderate level of variation (standard deviation). The control variables exhibit varying means and standard deviations, indicating diversity in the levels of openness, research and development investment, and foreign direct investment provinces. The descriptive statistics result indicates that the sample in our study can reflect the overall situation in China’s provinces.

**Table 4:** Descriptive statistics of variables

Variables	Definition	Mean	S.D	Min	Max
Dependent variable					
GTIE	Efficiency of green technology innovation in industrial enterprises	0.617	0.383	0.096	1.593
Independent variable					
ER	Local government environmental regulation	0.377	0.107	0.089	0.670
Control variables					
OPEN	Level of openness	0.288	0.338	0.017	1.698
TS	Research and development investment	0.020	0.014	0.004	0.072
FDI	Level of foreign direct investment	0.022	0.017	0.000	0.099

### Methodology

#### Spatial autocorrelation test

Given the interregional mobility of green finance and industrial enterprises’ green technological innovation, it is necessary to verify the spatial correlation among the samples. The first step is to conduct a spatial autocorrelation test using Moran’s *I* index. The Moran’s *I* index ranges from − 1 to 1. When the value falls within [− 1, 0) indicates negative spatial autocorrelation. A value of 0 suggests a random distribution pattern, while a value within (0, 1] indicates positive spatial autocorrelation. The closer the value is to absolute 1, the more pronounced the spatial clustering. The formula for calculating global spatial autocorrelation is as follows:

7 $$I=\frac{n{\sum }_{i=1}^{n}{\sum }_{j=1}^{n}{W}_{ij}({X}_{i}-\overline{X})({X}_{j}-\overline{X})}{{\sum }_{i=1}^{n}{\sum }_{j=1}^{n}{W}_{ij}{\sum }_{i=1}^{n}({X}_{i}-\overline{X}{)}^{2}}$$

In the formula, ${W}_{ij}$ represents the spatial weight matrix; ${X}_{i}$ and ${X}_{j}$ are the observed values at spatial locations *i* and *j*, respectively. $\overline{X}$ represents the average value of the geographic attribute observations.

#### Model building

The spatial panel econometric model incorporates spatial correlation into models and solves the bias problem in ordinary panel econometric models. Currently, there are three main types of spatial econometric models: spatial autoregressive model (SAR), spatial error model (SEM), and spatial Durbin model (SDM). Among them, the SDM is the basic form of spatial econometric models, which comprehensively considers the spatial interaction effects between the dependent variable and explanatory variables, as well as the spatial spillover effects of the local and neighboring areas. Therefore, this paper uses the SDM as the baseline model.

Given the theoretical assumptions mentioned earlier, it is important to investigate the impact of green finance on the efficiency of technological innovation in industrial enterprises, as well as the nonlinear moderating effects of local government environmental regulations. To investigate this, we first establish a baseline regression model without incorporating local government environmental regulations, denoted as model 1 in Eq. 8. Next, we introduce ER^2^ (square term for environmental regulation) and the interaction term between the centered GF (green finance) and ER^2^ to explore the moderating effect of local government environmental regulations. This is represented as model 2 in Eq. 9.

8 $${\mathrm{GTIE}}_{it}={\beta }_{0}+\delta {\mathrm{WGTIE}}_{it}+\beta {\mathrm{GF}}_{it}+\theta {\mathrm{WGF}}_{it}+{\gamma }_{j}{\mathrm{controls}}_{it}+{\mu }_{i}+{\eta }_{t}+{\varepsilon }_{it}$$

9 $${\mathrm{GTIE}}_{it}={\beta }_{0}+\delta {\mathrm{WGTIE}}_{it}+{\beta }_{1}{\mathrm{GF}}_{it}+\theta {\mathrm{WGF}}_{it}+{{{\beta }_{2}\mathrm{ER}}_{it}}^{2}+{\beta }_{3}({\mathrm{GF}}_{it}-\overline{{\mathrm{GF} }_{i}})\times {({\mathrm{ER}}_{it}-\overline{{\mathrm{ER} }_{i}})}^{2}+{\gamma }_{j}{\mathrm{controls}}_{it}+{\mu }_{i}+{\eta }_{t}+{\varepsilon }_{it}$$

Among them, $\overline{{\mathrm{GF} }_{i}}=\frac{1}{T}{\sum }_{t=1}^{T}{\mathrm{GF}}_{it}$, $\overline{{\mathrm{ER} }_{i}}=\frac{1}{T}{\sum }_{t=1}^{T}{\mathrm{ER}}_{it}$. $\delta$ represents the coefficient of spatial lagged dependent variable; $W$ represents the spatial weight matrix; ${\mathrm{WGTIE}}_{it}$ represents the spatial interaction effect among the dependent variables in the spatial econometric model. ${\mathrm{GF}}_{it}$ represents the main explanatory variable in the model; ${{\mathrm{ER}}_{it}}^{2}$ represents the moderating variable of local government environmental regulations with nonlinear effects; ${\mathrm{controls}}_{\mathrm{it}}$ represents the control variable; ${\beta }_{x}$ represents the estimated parameters or coefficients of the explanatory variables in the model; ${\gamma }_{j}$ represents the estimated coefficients of the control variables in the model.; $\mu$ represents the spatial-specific effects in the spatial econometric model; $\eta$ represents the time-specific effects in the econometric model; and $\varepsilon$ represents the random error term in the econometric model.

## Empirical analysis

### Spatial correlation analysis

**Fig. 1:**
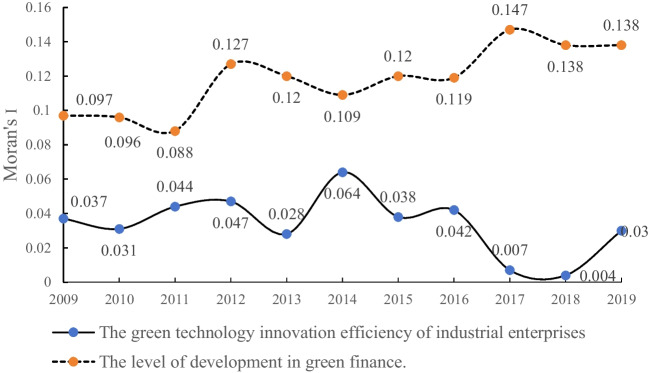
Overall Moran’s *I* index of green technology innovation efficiency and green finance development level of industrial enterprises from 2009 to 2019

Moran’s *I* index is a statistical measure used in spatial analysis and geostatistics to assess spatial autocorrelation, which is the degree to which the values of a variable at one location are correlated with the values of the same variable at neighboring locations. A positive Moran’s *I* indicates positive spatial autocorrelation, suggesting that neighboring spatial units tend to have similar values for the variable. In this study, we conducted a global spatial autocorrelation analysis using Moran’s *I* index to examine the spatial distribution patterns of green finance development and green technological innovation in 30 provinces in China from 2009 to 2019. We constructed a spatial weight matrix based on the actual distances between provinces, using the geographical distance between provincial capitals. The spatial weight matrix (*W*_*d*_) was defined as *W*_*d*_ = *1/d*_*ij*_, where *d*_*ij*_ represents the geographical distance between provincial capitals calculated using latitude and longitude data. The computed results are shown in the following figure:

Based on Fig. 1, it can be observed that the green finance development level and green technological innovation efficiency in the 30 provinces of China from 2009 to 2019 exhibit positive spatial correlation, indicating that these two variables have a spatial clustering effect and can be analyzed using a spatial econometric model. This suggests that higher levels of green finance development are clustered in regions with higher efficiency in green technological innovation. The Moran’s *I* index for the green technological innovation efficiency shows an overall “*N*”-shaped trend, initially increasing, then decreasing, and then increasing again. This indicates that the efficiency of green technological innovation is undergoing a reshaping process at the national level. Conversely, the Moran’s *I* index for the green finance development level demonstrates a gradual increase over time. This suggests that there is a growing spatial clustering of green finance resources, with these resources tending to concentrate in regions with higher levels of development. These results indicate the existence of spatial patterns and dynamics in the relationship between green finance development and green technological innovation efficiency across the provinces of China.

### Spatial panel econometric model testing

According to the spatial correlation analysis, there is significant spatial clustering in both the green finance development level and green technological innovation efficiency. Therefore, it is appropriate to use a spatial econometric model for regression analysis. In line with the testing procedure outlined by Elhorst (2014), we select a suitable spatial econometric model for this study.

Initially, we perform a mixed regression using the robust LM test, and the LM test results for both the SAR and SEM models reject the null hypothesis, indicating the appropriateness of both models. Therefore, we initially choose the SDM model that combines both of them. To determine whether to use fixed effects or random effects, we conduct a spatial Hausman test, and the results suggest that the spatial fixed effects model is more suitable for this study.

Next, we perform an LR fixed effects test on the spatial panel model and select the individual fixed effects SDM model among individual fixed effects, time fixed effects, and both fixed effects, based on the test results. To further validate the choice of the SDM model over SAR and SEM models, we estimate the SAR, SEM, and SDM spatial econometric models separately and conduct LR and Wald tests for model fit. The results of the LR and Wald tests also indicate that the SDM model is superior to the SAR and SEM models.

Furthermore, the overall goodness-of-fit of the SDM model is better than that of the SAR and SEM models, as evident from the regression results. Based on this, we choose the individual fixed effects SDM model to analyze the spatial effects of green finance development on the efficiency of green technological innovation. The specification test results for the model are shown in Table 5, and the regression results of the spatial econometric model are presented in Table 6.

**Table 5:** Specification test of spatial econometric models

Dependent variable	Statistics	LM hypothesis testing		LR hypothesis testing		Wald hypothesis testing	
		LM value	*p*-value	LR value	*p*-value	Wald value	*p*-value
GTIE	Spatial-lag	4.100	0.0429	7.62	0.0058	11.65	0.0006
	Spatial-error	3.867	0.0493	9.56	0.0020	7.53	0.0061

**Table 6:** Results of the spatial econometric regression

Independent variable	OLS	SAR		SEM		SDM	
		Model 1	Model 1	Model 1	Model 2	Model 1	Model 2
GF	1.307^***^ (4.58)	0.941^***^ (3.56)	1.007^***^ (3.75)	0.761^***^ (2.63)	0.827^***^ (2.84)	0.657^**^ (2.33)	0.730^**^ (2.56)
ER^2^			176.214^**^ (2.10)		184.808^**^ (2.11)		169.147^**^ (2.03)
GF x ER^2^			− 920.711^**^ (− 2.24)		− 949.005^**^ (− 2.27)		− 895.555^**^ (− 2.19)
OPEN	0.225^*^ (1.96)	0.260^**^ (2.50)	0.291^***^ (2.80)	0.296^***^ (2.59)	0.325^***^ (2.84)	0.377^***^ (3.38)	0.405^***^ (3.63)
TS	6.617^***^ (2.82)	4.913^**^ (2.31)	5.363^**^ (2.52)	4.397^*^ (1.93)	4.905^**^ (2.15)	3.336 (1.52)	3.797^*^ (1.73)
FDI	− 0.409 (− 0.38)	0.830 (0.84)	0.686 (0.70)	1.505 (1.47)	1.372 (1.34)	1.753^*^ (1.68)	1.592 (1.54)
W $\times$ GF						1.903^***^ (2.74)	1.864^***^ (2.70)
$\delta /\lambda$		0.508^***^ (5.75)	0.508^***^ (5.76)	0.564^***^ (6.25)	0.565^***^ (4.91)	0.367^***^ (3.34)	0.369^***^ (3.37)
*R*-sq	0.3869	0.4170	0.4233	0.4324	0.4361	0.4374	0.4427

^***^, ^**^, and ^*^ indicate significance at the 0.01, 0.05, and 0.10 levels, respectively; *t*-values are shown in parentheses. The same notation applies to Tables 6, 7, and 8

### The empirical results of the spatial econometric model

Based on the findings presented in Table 6, in model 1, it can be concluded that the level of green finance development has a significant positive impact on the green technology innovation efficiency of industrial enterprises in the spatial Durbin model (SDM), confirming hypothesis 1. To further explore the nonlinear moderating effect of local government environmental regulation, a squared term of local government environmental regulation and an interaction term between the squared term of green finance development and local government environmental regulation were added. The regression results of model 2 show that the interaction term between heterogeneous local environmental regulation and the level of green finance development in the SDM has a negative regression coefficient on industrial enterprise green technology innovation, and it passes the significance test at the 5% level. This suggests that local environmental regulation plays a “inverted U-shaped” moderating role in the relationship between green finance development and the efficiency of industrial enterprise green technology innovation, confirming hypothesis 3.

The empirical findings demonstrate that green finance in China plays a significant role in supporting the green technology innovation of industrial enterprises. An efficient green finance system offers capital support to enterprises, enabling them to invest more effectively in environmental innovation. This is consistent with resource dependency theory, indicating that enterprises, through effective financial support, are better equipped to address environmental challenges and drive the implementation of green technology innovations. The positive impact of green finance on industrial enterprise green technology innovation efficiency also varies under different levels of local government environmental regulation. When the level of environmental regulation is low, its improvement can facilitate the deepening of green finance in industrial enterprise green technology innovation. Moderate regulation by local government environmental regulation helps to create a virtuous cycle between green finance and industrial enterprise green technology innovation. However, once local government environmental regulation exceeds the critical threshold, its improvement will instead inhibit the promotion of deepening green finance, and the excessive environmental regulation will squeeze the support of green finance for industrial enterprise green technology innovation. This conclusion aligns with sustainable development theory and environmental economics, emphasizing the potential for a virtuous cycle between environmental regulations and green finance. Moderate local government environmental regulations contribute to an intrinsic environmental commitment by enterprises. When coupled with financial support from green finance, the two factors complement each other. This cycle strengthens the commitment of enterprises to environmental innovation, aiding in achieving sustainable development goals.

Because the regression coefficient of SDM model does not explain the spatial action mechanism of green finance development on green technology innovation of industrial enterprises from three aspects of local, peripheral, and total effect, the decomposition effects of each influencing factor are calculated separately, as shown in Table 7.

**Table 7:** Spatial effects decomposition results of the SDM model

Independent variable	Direct effect		Indirect effect		Total effect	
	Model 1	Model 2	Model 1	Model 2	Model 1	Model 2
GF	0.726^**^ (2.53)	0.799^***^ (2.76)	3.337^***^ (3.84)	3.350^***^ (3.56)	4.063^***^ (4.68)	4.148^***^ (4.40)
ER^2^		167.761^**^ (2.07)		98.722 (1.48)		266.483^*^ (1.95)
GF x ER^2^		− 884.365^**^ (− 2.20)		− 520.583 (− 1.57)		− 1404.947^**^ (− 2.08)
OPEN	0.377^***^ (3.48)	0.421^***^ (3.93)	0.230^*^ (1.78)	0.246^**^ (2.10)	0.607^***^ (3.05)	0.667^***^ (3.56)
TS	3.612^*^ (1.70)	3.793^*^ (1.77)	2.281 (1.19)	2.213 (1.36)	5.894 (1.57)	6.005^*^ (1.70)
FDI	1.724^*^ (1.71)	1.621 (1.63)	1.073 (1.24)	0.964 (1.24)	2.797 (1.59)	2.585 (1.56)

**Table 8:** Robustness test estimation results

Independent variable	OLS		SDM (*W*)		SDM (*W*_*e*_)	
	Model 3	Model 4	Model 1	Model 2	Model 1	Model 2
GF	1.307^***^ (4.58)	1.360^***^ (4.68)	0.831^***^ (2.99)	0.884^***^ (3.13)	0.474^*^ (1.68)	0.554^*^ (1.94)
ER^2^		190.447^**^ (2.04)		160.800^*^ (1.89)		157.742^*^ (1.92)
GF x ER^2^		− 944.591^**^ (− 2.06)		− 797.455^*^ (− 1.92)		− 865.853^**^ (− 2.15)
OPEN	0.225^*^ (1.96)	0.257^**^ (2.22)	0.316^***^ (2.92)	0.343^***^ (3.15)	0.315^***^ (2.95)	0.343^***^ (3.20)
TS	6.617^***^ (2.82)	7.117^***^ (3.03)	4.410^**^ (2.03)	4.873^**^ (2.24)	3.747^*^ (1.77)	4.138^*^ (1.95)
FDI	− 0.409 (− 0.38)	− 0.546 (− 0.51)	1.045 (1.02)	0.901 (0.88)	1.184 (1.21)	1.028 (1.05)
W x GF			1.161^***^ (2.65)	1.162^***^ (2.67)	1.654^***^ (3.83)	1.625^***^ (3.77)
$\delta$			0.248^***^ (3.70)	0.237^***^ (3.53)	0.288^***^ (3.76)	0.291^***^ (3.80)
*R*-sq	0.3869	0.3956	0.4306	0.4408	0.4310	0.4421
Direct effect						
GF			0.922^***^ (3.28)	0.973^***^ (3.41)	0.605^**^ (2.12)	0.685^**^ (2.39)
ER^2^				159.714^*^ (1.93)		157.801^*^ (1.95)
GF x ER^2^				− 786.793^*^ (− 1.92)		− 862.858^**^ (− 2.16)
Indirect effect						
GF			1.726^***^ (3.43)	1.735^***^ (3.14)	2.394^***^ (4.98)	2.405^***^ (4.49)
ER^2^				46.606 (1.58)		60.665 (1.54)
GF x ER^2^				− 228.535 (− 1.59)		− 331.727^*^ (− 1.68)
Total effect						
GF			2.648^***^ (4.75)	2.707^***^ (4.43)	2.999^***^ (5.73)	3.090^***^ (5.32)
ER^2^				206.320^*^ (1.91)		218.466^*^ (1.90)
GF x ER^2^				− 1015.337^*^ (− 1.91)		− 1194.585^**^ (− 2.11)

According to the spatial effect decomposition results of the SDM model, both the direct and indirect effects of the level of green finance development on the efficiency of industrial enterprise green technology innovation are significantly positive. This indicates that the current level of green finance development not only promotes local industrial enterprise green technology innovation but also has significant spatial spillover effects on the green technology innovation of industrial enterprises in neighboring areas, confirming hypothesis 2.

Upon examining the magnitude of the direct and indirect effects of green finance development, it becomes evident that the promotion effect of green finance development on the green technology innovation efficiency of industrial enterprises in neighboring areas is significantly greater than that on the local area. The liquidity of green finance significantly drives the green technology innovation of industrial enterprises in neighboring areas.

Further analysis of the nonlinear moderating effect of environmental regulation reveals that the overall spatial effect and direct effect of the interaction term (GF x ER^2^) representing the nonlinear regulatory effect of environmental regulation are significant. However, the indirect effect is not significant, implying that the regulatory effect of local government environmental regulation still focuses on the impact of local green finance development on industrial enterprise green technology innovation, with priority given to local development, and the spillover effect on neighboring areas is not apparent.

### Robustness test

#### Moderation effects in panel data

Previous studies have primarily focused on investigating the nonlinear moderation effects of local government environmental regulations on the relationship between the level of green finance development and the efficiency of green technology innovation in industrial enterprises using the spatial Durbin model. To further test the robustness of the results regarding the nonlinear moderation effects, we employ a panel data fixed effects model based on the ordinary least squares (OLS) method. The baseline regression model for panel data is specified as model 3, as shown in Eq. 10, while the model for nonlinear moderation effects in panel data is specified as model 4, as shown in Eq. 11.

10 $${\mathrm{GTIE}}_{it}={\beta }_{0}+\beta {\mathrm{GF}}_{it}+{\gamma }_{j}{\mathrm{controls}}_{it}+{\varepsilon }_{it}$$

11 $${\mathrm{GTIE}}_{it}={\beta }_{0}+{\beta }_{1}{\mathrm{GF}}_{it}+{{{\beta }_{2}\mathrm{ER}}_{it}}^{2}+{\beta }_{3}({\mathrm{GF}}_{it}-\overline{{\mathrm{GF} }_{i}})\times {({\mathrm{ER}}_{it}-\overline{{\mathrm{ER} }_{i}})}^{2}+{\gamma }_{j}{\mathrm{controls}}_{it}+{\varepsilon }_{it}$$

Regression results of model 3 and model 4 are shown in Table 7; furthermore, the level of green finance development has a positive impact on the green technological innovation efficiency of industrial enterprises in the panel data regression. Furthermore, the non-linear moderation effect of local government environmental regulation still exhibits an inverted U-shaped pattern after introducing the non-linear moderating variable. This is consistent with the estimation results of the spatial econometric model, indicating the robustness of the promotion effect of green finance and the non-linear moderation effect of local government environmental regulation.

#### Set different spatial weight matrices

The choice of different spatial weight matrices can lead to significantly different regression results in spatial econometric models. Therefore, this study continues to construct adjacency spatial weight matrix (*W*) and economic distance spatial weight matrix (*W*_*e*_) to replace the geographic distance spatial weight matrix (*W*_*d*_) in order to test the stability of the results. The adjacency spatial weight matrix is set as 1 if two provinces are adjacent, and 0 otherwise. The economic distance spatial weight matrix is defined as ${W}_{e}=1/\left|{\overline{Q} }_{i}-{\overline{Q} }_{j}\right|$, ${\overline{Q} }_{i}$ and ${\overline{Q} }_{j}$ denote the average per capita GDP of province *i* and province *j* during the period of 2009–2019, respectively. Even after using the *W* and *W*_*e*_ spatial weight matrices, the SDM model remains the optimal spatial econometric model in this study. The results show that although the coefficients in the regression results may vary slightly compared to the previous findings, the direction and significance levels of the coefficients remain fundamentally unchanged, confirming the robustness and reliability of the main regression results in this spatial econometric analysis.

## Conclusion and policy recommendations

### Conclusion

This study combines the current status of green finance development in China using a sample of 30 provinces from 2009 to 2019. To evaluate the level of green finance development, the study employs the subjective and objective weighting method. The efficiency of industrial enterprise green technology innovation is measured using the super-efficiency SBM model with undesirable outputs. The impact of green finance development on industrial enterprise green technology innovation efficiency and its spatial effects are explored using the spatial Durbin model. Furthermore, the study incorporates the square term of local government environmental regulation to investigate its nonlinear moderating effect on the relationship between green finance development and industrial enterprise green technology innovation. The research findings are as follows: First, green finance serves as a financial regulator, policy guide, and consultant, and has a positive effect on promoting the efficiency of industrial enterprise green technology innovation. Second, local government environmental regulation has a nonlinear “inverted U-shaped” moderating effect on the relationship between green finance development and industrial enterprise green technology innovation. Appropriate environmental regulation effectively incentivizes the coordinated development of green finance and industrial enterprise green technology innovation. However, excessive local government environmental regulation significantly squeezes the profit margins of financial institutions and industrial enterprises, leading to a negative moderating effect on the impact of green finance on industrial enterprise green technology innovation. Third, in terms of spatial effects, the level of green finance development has significant positive direct spatial effects and spatial spillover effects on the green technology innovation of industrial enterprises. The regional connectivity function of green finance is highlighted. Meanwhile, the spatial spillover effects of local government environmental regulation and their heterogeneous moderating effect vary significantly under different spatial weight matrices. Local governments tend to prioritize local development when implementing green development policies, and the regulatory capacity for green finance and industrial enterprise green technology innovation in neighboring areas still needs improvement.

### Policy recommendations

The research conclusions of this study confirm the spatial effects of green finance development and industrial enterprise green technology innovation efficiency under local government environmental regulation. The study provides some policy implications: First, strengthen the ability of green finance to support the green transformation of industrial enterprises. Continuously increase investment in green finance based on the current level of green finance development, innovate green finance products, mobilize multiple departments to participate in green investment and financing processes, make the green finance service environment more efficient and transparent, and establish a more comprehensive green finance service system. Second, enhance the endogenous regulatory capacity of local government environmental regulation on the green finance market. While adhering to the top-level design of the central government, local governments need to consider local resource endowments and industrial characteristics. They should proactively improve the regulatory capacity of green finance policy formulation and industrial enterprise green innovation based on local practices, fully mobilize the subjective initiative of financial institutions and industrial enterprises, integrate incentive mechanisms and constraint mechanisms, and avoid excessive administrative penalties and intervention in the green finance market and industrial enterprises’ pollution behavior. Third, enhance the regional connectivity function of green finance and develop a nationally interconnected local environmental regulation system. While supporting the flow of green funds across regions, utilize corresponding financial technology to promote the disclosure and sharing of information on industrial enterprises’ pollution and green innovation behaviors. For example, connect the comprehensive “green finance + big data” service platforms established in various regions to overcome the “information silo” between financial institutions and industrial enterprises across regions. While local governments formulate supporting policies to foster local advantages in green industries, they should also collaborate with neighboring regions to issue corresponding guidelines with a focus on economic belts and urban clusters. They should improve the green guarantee mechanisms and dynamic monitoring mechanisms of green finance, promote open cooperation among local governments, establish environmental credit evaluation and pollution enterprise information disclosure systems, achieve coordinated cooperation between green industrial policies and green finance and green industry development, and establish a nationwide interconnected green supply chain management cooperation framework.

### Limitations and future research directions

This paper investigates the impact of green finance and the effect of green finance on the efficiency of green technological innovation of industrial enterprises under the moderating effect of local government environmental regulations. However, it still suffers from the following limitations that could be strengthened by future research. First, the study focuses on China, and while it provides valuable insights for that context, the generalizability of the findings to other countries or regions may be limited. Future research could conduct comparative studies across multiple countries or regions to assess the generalizability of the findings and explore the impact of different regulatory environments and financial systems on green finance and technology innovation. Second, the study confirms the nonlinear moderating effect of local government environmental regulation on green finance and green technology innovation of industrial enterprises. Nevertheless, the study interval is still short, and the impact of green finance on green technological innovation can be tracked over time to understand how these relationships evolve over time.

## Footnotes

1 Source of information: European Commission: https://ec.europa.eu/info/business-economy-euro/banking-and-finance/sustainable-finance_en
